# The longevity-associated BPIFB4 gene guarantees vascular homeostasis and immune protection through platelets

**DOI:** 10.1007/s11357-024-01242-9

**Published:** 2024-06-17

**Authors:** Elena Ciaglia, Francesco Montella, Albino Carrizzo, Valentina Lopardo, Roberta Maria Esposito, Cristina Basile, Antonio Damato, Massimiliano De Lucia, Anna Maciag, Gaia Spinetti, Maria Serena Milella, Davide Maselli, Carmine Vecchione, Annibale Alessandro Puca

**Affiliations:** 1 Department of Medicine, Surgery and Dentistry “Scuola Medica Salernitana”, University of Salerno, Via Salvatore Allende, 84081 Baronissi Salerno, Italy; 2 Vascular Physiopathology Unit, IRCCS Neuromed, Pozzilli, Italy; 3 Cardiovascular Research Unit, IRCCS MultiMedica, 20138 Milan, Italy

**Keywords:** BPIFB4 gene, Vascular homeostasis, Platelets

## Abstract

Beyond their activity in hemostasis and thrombosis, recent advances attribute platelets a pro-youthful role capable to attenuate immune senescence and age-related neuroinflammation. Previous studies from our group associated a polymorphic haplotype variant in the BPIFB4 gene (LAV-BPIFB4) with exceptional longevity. Transfer of the LAV-BPIFB4 in preclinical models has proved strategic to cope with frailty conditions, aging-related events, e.g., cardiovascular ones, and immune dysfunction mainly through a favorable conditioning of the immune system. However, whether platelets participate in LAV-BPIFB4 therapeutic action is currently unknown. Herein, we discovered that platelets were instrumental in boosting the favorable health outcomes of the systemic AAV-LAV-BPIFB4 gene transfer *in vivo*, as the α-CD42b platelet depletion completely abolished the vascular protective action of LAV-BPIFB4 and suppressed its pro-resolutive CD206 + anti-/CD86 + pro-inflammatory Ly6C + monocyte skewing to LPS stimulation. Of note, this is associated with a huge drop in the protective levels of BPIFB4 in the plasma of AAV-LAV-BPIFB4-injected C57BL/6 mice, indicating that plasma circulating platelets may be a reservoir of the BPIFB4 protein. Indeed, we noticed that BPIFB4 was released by human platelets, a process that is amplified in LAV-allele carrier donors. Accordingly, lentivirus-mediated overexpression of human LAV-BPIFB4 isoform, but not WT-BPIFB4 isoform was able in leading differentiated megakaryocytes to release more platelet-like-particles enriched for BPIFB4. In addition, *in vitro*, the M2 macrophage polarization increased when releasate from platelets, and even more from LAV pre-stimulated once, was added in monocyte cell culture. Our data suggest that platelet release of BPIFB4 and of yet-to-be-determined unidentified factors mediates the therapeutic efficacy of LAV-BPIFB4 treatment.

**Supplementary Information:**

The online version contains supplementary material available at 10.1007/s11357-024-01242-9.

## Introduction

Platelets are figurative elements of the blood, anucleate, with the primary function of mediating the coagulation process and hemostasis [1]. In recent years, the role of platelets has widened with discovery of evidence supporting their ability to finely tune the inflammatory response, in particular the innate immune arm [2]. Likewise, platelets can regulate fundamental biological processes ranging from wound healing to neurogenesis through the regulated release of their protein content. Notably, platelet factors are induced by longevity factor klotho, or just upon physical activity to enhance cognitive function both in aged and young mice [3–5]. Thus, it is interesting to speculate that platelets may serve as pro-youthful messengers capable to counteract age-related decline.

In the past few years, we extensively documented the peculiar activity of a longevity-associated variant (LAV) of bactericidal/permeability-increasing fold-containing family-B-member-4 (BPIFB4) protein encoded by a four-SNP haplotype of BPIFB4 gene in promoting health/longevity. Over time *LAV-BPIFB4* gene therapy has demonstrated anti-atherosclerotic [6], anti-hypertensive [7, 8], pro-angiogenic [7], and neuroprotective activities [9], improved frailty indices [10], rescued diabetic cardiomyopathy [11], and delayed cardiac aging [12] and ischemic heart dysfunctions [13], mainly by counterbalancing chronic inflammation both in vivo and in ex vivo [6]. The enhanced qualities of the LAV-BPIFB4 isoform, compared to WT-BPIFB4 isoform, and its association with higher BPIFB4 protein levels in the plasma, raised suspicion about the existence of a circulating cellular reservoir that might be a rapid source of BPIFB4 protein and its prognostic relevance. Thus, we wondered if platelets could participate in LAV-BPIFB4 therapeutic action and if BPIFB4 could serve as a favorable platelet factor. This will be an important knowledge advance as the abundance of BPIFB4 in the serum of Long Living Individuals (LLIs) was primarily associated with their ability to maintain a healthy balance between anti-inflammatory and pro-inflammatory mechanisms without experiencing the adverse immune system remodeling typically associated with aging [14]. Circulating levels of BPIFB4 were also favorably associated with a higher frequency of nonclassical patrolling monocytes [15], which are endowed with reparative and proangiogenic action. Furthermore, BPIFB4 has been proven to be indispensable in skewing macrophage response toward a pro-resolving M2-state both in microglia and atherosclerotic plaque [6, 9]. Therefore, the protein carrier role of platelets and their contribution to rejuvenation prompted us to investigate the involvement of platelets in the immune modulatory role of the BPIFB4 protein and in the functional effects of the (rh)LAV-BPIFB4 *in vitro*, and the AAV-LAV-BPIFB4 constructs *in vivo*.

## Results

### Platelets contribute to the pool of BPIFB4 in plasma and to its effects on both vascular reactivity and monocyte inflammatory profile

**Fig. 1:**
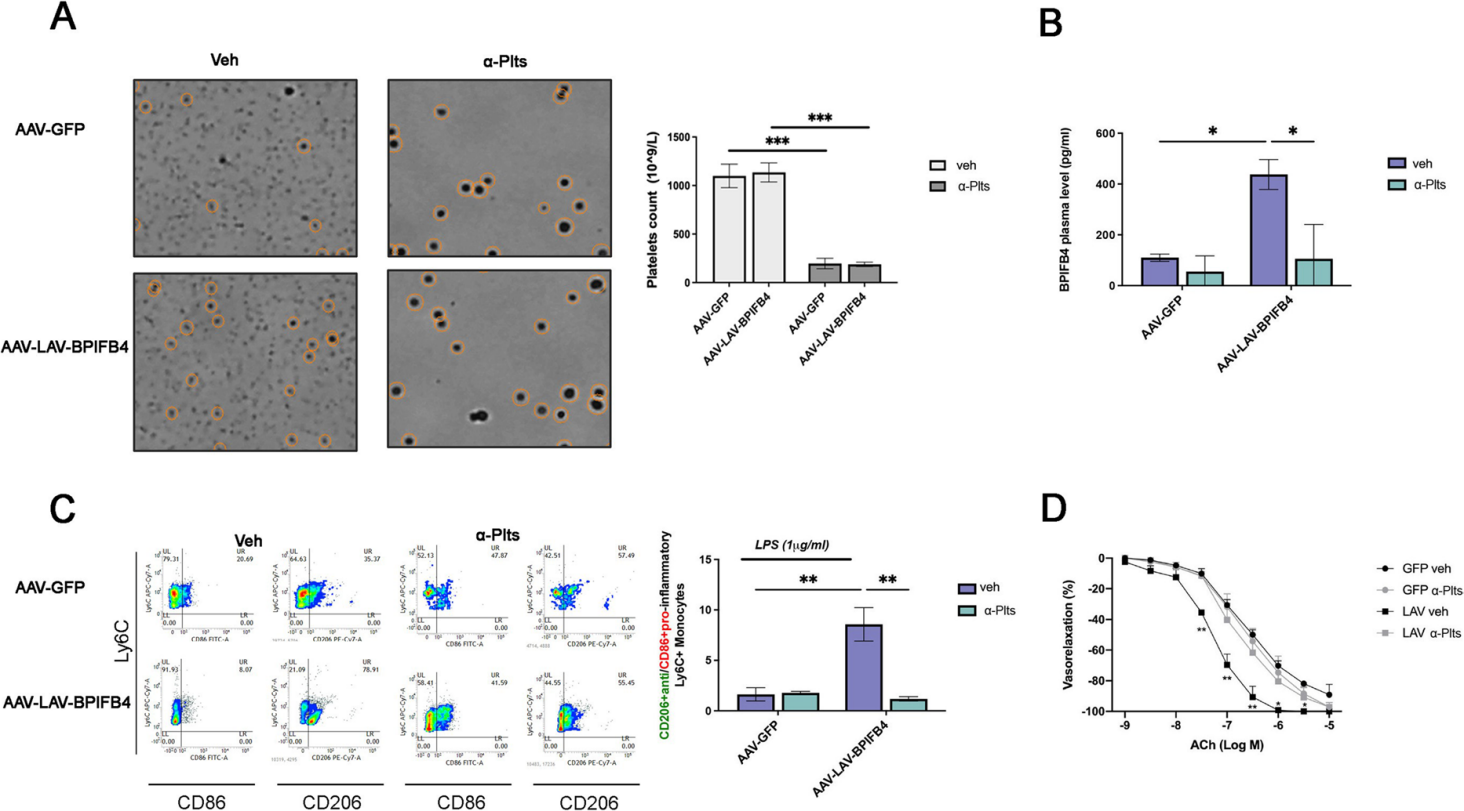
Platelets route the protective and immunomodulatory effects of BPIFB4. **A** Platelets count in *n* = *4* AAV-GFP-ISO (AAV-GFP vehicle), *n* = *4* anti-platelets (anti-GPIbα)-treated AAV-GFP (AAV-GFP α-Plts), *n* = *4* AAV-LAV-BPIFB4 (AAV-LAV vehicle), and *n* = *4* anti-platelets (anti-GPIbα)-treated AAV-LAV-BPIFB4 (AAV-LAV α-Plts) mice. The square boxes on the left are representative cell count images, showing the efficient platelets depletion as indicated in the graph bar on the right. **B** The bar graph shows the BPIFB4 plasma levels (pg/mL) of *n* = *4* AAV-GFP-ISO (AAV-GFP vehicle), *n* = *4* anti-platelets (anti-GPIbα)-treated AAV-GFP (AAV-GFP α-Plts), *n* = *4* AAV-LAV-BPIFB4 (AAV-LAV vehicle), and *n* = *4* anti-platelets (anti-GPIbα)-treated AAV-LAV-BPIFB4 (AAV-LAV α-Plts) mice. Platelet depletion minimizes the BPIFB4 release characterizing AAV-LAV-BPIFB4 mice. **C** The data shows quantification of the CD206 + /CD86 + ratio (indicative of the anti-inflammatory or pro-inflammatory-like monocyte phenotype) among total circulating Ly6C + monocytes in *n* = *4* AAV-GFP-ISO (AAV-GFP vehicle), *n* = *4* anti-platelets (anti-GPIbα)-treated AAV-GFP (AAV-GFP α-Plts), *n* = *4* AAV-LAV-BPIFB4 (AAV-LAV vehicle), and *n* = *4* anti-platelets (anti-GPIbα)-treated AAV-LAV-BPIFB4 (AAV-LAV α-Plts) mice. Murine PBMCs were treated with LPS 1 µg/mL for 24 h, harvested, and acquired by using FACS Verse Flow Cytometer (BD Biosciences). AAV-LAV-BPIFB4 infection provides an anti-inflammatory monocyte phenotype that is missed due to platelet depletion. On the left is a representative FACS dot plot analysis of the results expressed with the bar graph on the right side. Results from each treatment group are expressed as mean ± SD. Pairwise comparisons statistically significant are indicated (two-way ANOVA followed by Tukey’s multiple comparisons test, **p < 0.01). **D** Vascular response of ex vivo mesenteric arteries from *n* = *4* AAV-GFP-ISO (AAV-GFP vehicle), *n* = *4* anti-platelets (anti-GPIbα)-treated AAV-GFP (AAV-GFP α-Plts), *n* = *4* AAV-LAV-BPIFB4 (AAV-LAV vehicle), and *n* = *4* anti-platelets (anti-GPIbα)-treated AAV-LAV-BPIFB4 (AAV-LAV α-Plts) mice. The curves show the dose–responses to acetylcholine (Ach) after 4 days of AAV- treatment prior to platelets depletion. AAV-GFP was used as a control. Values are mean ± standard deviation of four independent experiments. Platelet depletion reduces the LAV-BPIFB4 effect on Ach vasorelaxation. Results are expressed as mean ± SD. Pairwise comparisons statistically significant are indicated (two-way ANOVA followed by Tukey’s multiple comparisons test, **p < 0.01)

Previous studies have shown that high BPIFB4 expression can be achieved after LAV-BPIFB4 gene transfer and that it may account for most of LAV-BPIFB4 beneficial effects. Notably, sustained BPIFB4 levels were achieved in plasma. Because of the role of platelets as a key circulating source of biological factors in plasma, we asked if platelets could be a reservoir of BPIFB4 and a means of LAV-BPIFB4 activity. To this end, C57BL/6 (wild-type) mice were divided into two groups and treated with either a platelet blocking antibody α-CD42b (α-Plts) or a control antibody (veh) 4 days after LAV-BPIFB4 gene or control GFP delivery using adeno-associated viral (AAV) vectors. Antibody-mediated platelets depletion resulted in a > 80% reduction in circulating platelets count (1098 ± 121 × 10^9^/l versus 197 ± 58 × 10^9^/l in antibody control-treated mice) (Fig. 1A). As expected, in platelet-competent mice, the BPIFB4 plasma levels after 4 days of treatment with AAV-LAV-BPIFB4 significantly increased as compared to AAV-GFP-treatment based on an enzyme-linked immunosorbent assay (ELISA). On the contrary, we detected reduced levels of BPIFB4 in the blood plasma preparations from AAV-LAV-BPIFB4 treated/platelet-depleted mice (Fig. 1B), mainly indicating a putative participation of platelets as a cellular source of BPIFB4 protein amount. Next, we wondered if platelets contribute to both vascular and immunomodulatory effects by LAV-BPIFB4.

In platelet-competent mice, the blood monocyte CD206 + anti-/CD86 + pro-inflammatory Ly6C + ratio after *ex vivo* LPS stimulation was significantly elevated in AAV-LAV-BPIFB4-treated group as compared to AAV-LAV-BPIFB4-treated/platelet-depleted mice (Fig. 1C), demonstrating the critical role of platelets related to the well-known anti-inflammatory effects of AAV-LAV-BPIFB4 treatment. Further, platelet-competent AAV-LAV-BPIFB4-mice were associated with an improvement of the vascular reactivity of ex vivo mesenteric arteries to acetylcholine. In contrast, in vivo platelet depletion completely abolished this beneficial effect (Fig. 1D).

### BPIFB4 content in human platelets

**Fig. 2:**
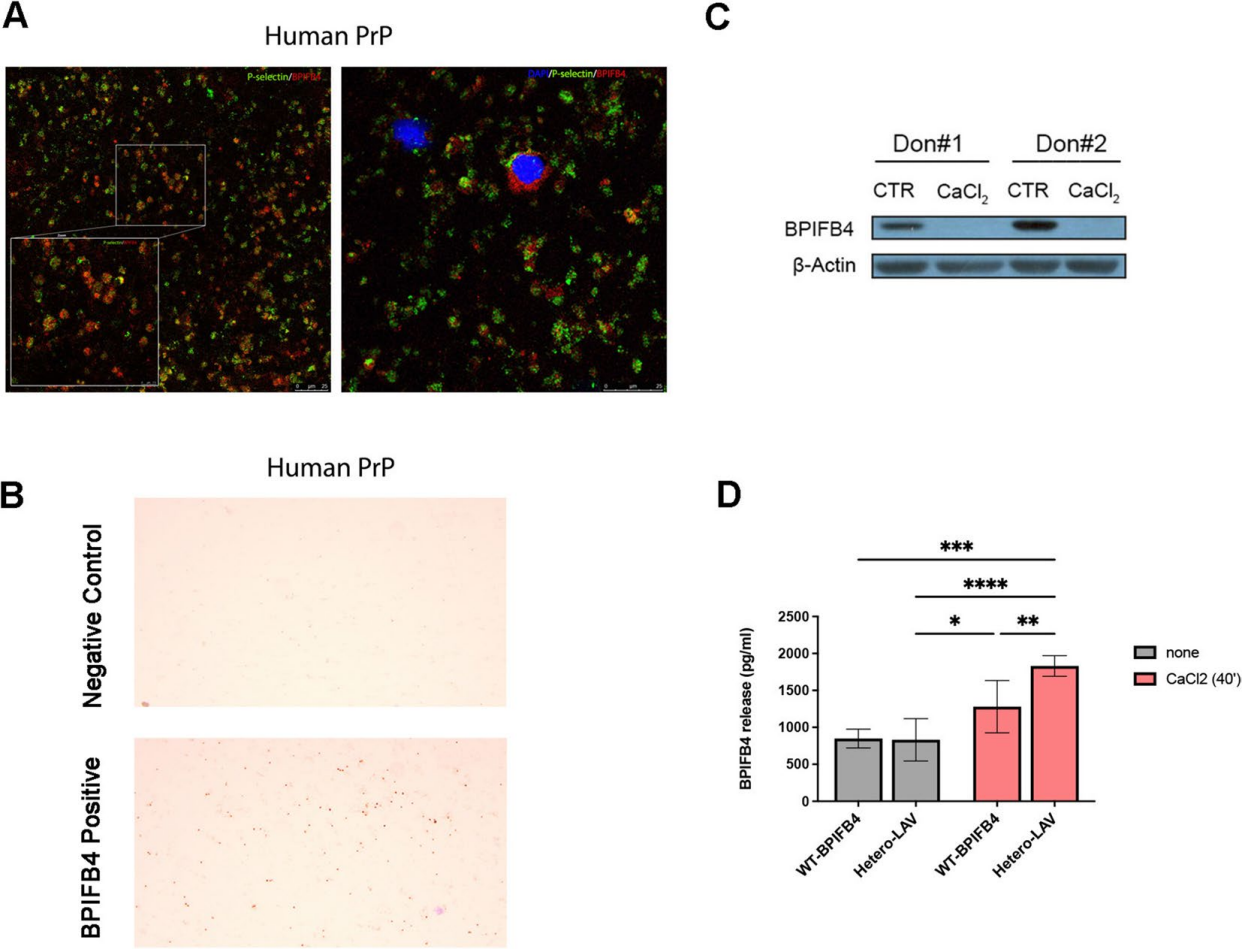
BPIFB4 expression profile in platelets from human platelet-rich plasma. **A** Representative confocal immunofluorescence staining of BPIFB4 in platelet from human platelet-rich plasma (PRP). PRP slides (≈10^4^ cells) were immunostained for image acquisition in three separate channels: DAPI (405 nm/nucleus), P-selectin (488 nm/green), and BPIFB4 (549 nm/red). Representative merged images are shown, magnification × 63. Platelets were identified based on their size, staining with P-selectin, and absence of nuclear DAPI stain. On the left, the image shows P-selective positive platelet co-expressing BPIFB4. Scale bar, 25 µm. Inset shows higher magnification, zoom × 3.46. Scale bar, 7.5 µm. On the right, platelets (P-selective positive cells) and cells stained with DAPI and BPIFB4. Scale bar 25 µm, zoom × 1.79. **B** The BPIFB4 platelets localization was shown in platelets from human PRP subjected to immunohistochemistry staining against BPIFB4 and acquired by using bright-field microscopy, at 10 × magnification. Negative control was obtained using only secondary antibodies staining. **C** Representative immunoblot bands of control and treated (CaCl_2_ 22 mM for 40 min) PRP from two donors immunoblotted for BPIFB4 and β-actin. Overall, the panel highlights the resting condition expression of BPIFB4 in platelets and the increase of the BPIFB4 release upon platelets’ activation. Pairwise comparison is reported (*t*-test; ****p < *0.0001).* **D** Bar graph shows BPIFB4 secretion levels (pg/mL) in *n* = 17 donors’ PRP in resting condition (grey bars) and upon 40 min CaCl_2_ 22mM treatment (red bars) detected by ELISA. PRPs were genotyped and stratified into *n* = 8 WT-BPIFB4 carrier and *n* = 9 LAV- and Hetero-BPIFB4 carrier donors. Pairwise comparisons are reported (two-way ANOVA followed by Tukey’s multiple comparisons test; *p < 0.05, **p < 0.01, ***p < 0.001, ****p < *0.0001*)

To investigate the putative extent and expression pattern of BPIFB4 in the platelets, we stained human platelets with fluorescent-labeled antibodies against a common platelet marker, P-selectin, and BPIFB4 (Fig. 2A). Double-label immunofluorescence confocal microscopy on fixed and permeabilized platelets revealed a large intracellular localization of BPIFB4. The wide localization of BPIFB4 in platelets was also confirmed immunohistochemically where fixed platelets showed increased positive staining to BPIFB4 (Fig. 2B) as compared to negative control. Likewise, Western Blot analysis of washed platelets from human PRP revealed that resting human platelets contained high levels of BPIFB4; stimulation with CaCl_2_(22 mM), used to induce massive platelet emptying, resulted in total disappearance in BPIFB4 levels in platelet lysates (Fig. 2C) which is consistent with the higher release of BPIFB4 in platelets supernatant (Fig. 2D). Interestingly, analysis of human genotyped PRP showed that LAV-BPIFB4 carriers released higher levels of BPIFB4 after platelet activation when compared with no-carriers (Fig. 2D).

### BPIFB4 expression: from myeloid progenitors to platelet release

**Fig. 3:**
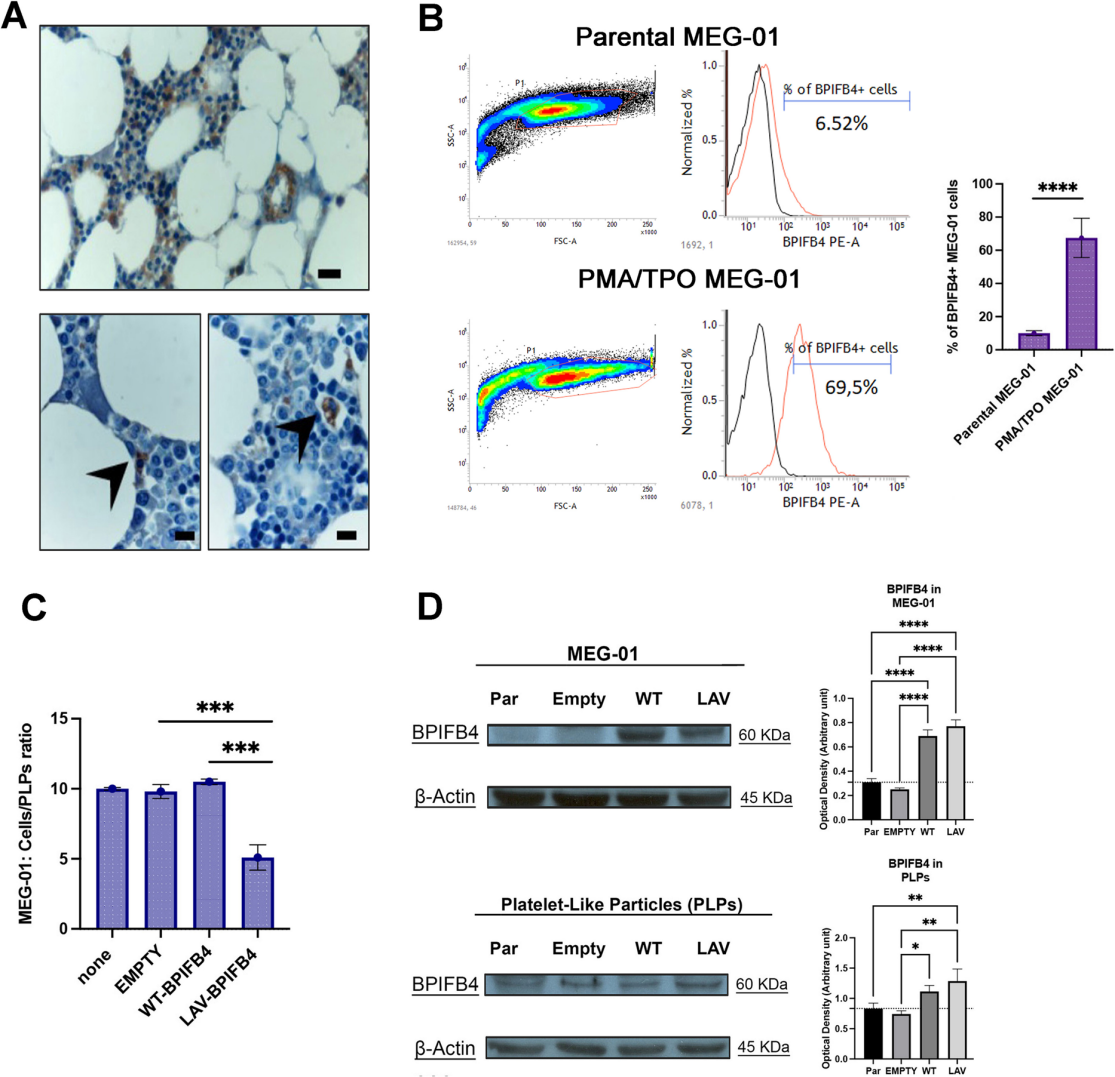
Characterization of BPIFB4 expression in naive and mature megakaryocytes. **A** Representative microphotographs of bone marrow labeled for BPIFB4 and DAB staining. Scale bar, 20 µm. At higher magnification: Common Myeloid Progenitor and Megakaryoblast. Scale bar, 10 µm. **B** Representative flow cytometry analysis of BPIFB4 expression in undifferentiated and PMA/TPO treated MEG-01 cells. On the right, the related bar graph shows the percentage of positive BPIFB4 + MEG-01 cells in naive and differentiated counterparts. Upon differentiation, MEG-01 cells expose higher amount of surface BPIFB4. The result is representative of 3 independent experiments expressed as mean ± SD. A pairwise comparison is reported (ordinary one-way ANOVA; ***p < *0.001).* **C** MEG-01 cells were infected with EMPTY lentiviral vector or particles encoding WT- or LAV-BPIFB4. Differentiated MEG-01 and platelet-like particles (PLPs) were counted by using an automatic cell counter (LUNA Automated Cell counter, logos) and considering two cell compartments: platelet-like particles, Ø 1–3 µm, and megakaryocytes, Ø 16–35 µm. LAV-BPIFB4-infected MEG-01 cells show lower ratio with respect to parental, EMPTY- and WT-BPIFB4 MEG-01 cells. The result is representative of 3 independent experiments expressed as mean ± SD. Pairwise comparisons are reported (ordinary one-way ANOVA; ***p < *0.001).* **D** On the left, representative western blot analysis of BPIFB4 in the different sets of MEG-01 cells and the corresponding PLPs. β-Actin was used as a control for quantitation of sample protein as summarized in densitometric analysis. On the right, the related bar graph of BPIFB4 quantification is shown. Pairwise comparisons are reported (ordinary one-way ANOVA; *p < 0.05, **p < 0.01, ****p < *0.0001)*

To determine whether BPIFB4 expression in platelets could derive from megakaryocytes, we explored the putative expression of BPIFB4 in the different megakaryopoiesis stages’ cell types. First, immunohistochemical staining of human bone marrow biopsies (Fig. 3A) showed that a small percentage of myeloid bone marrow cells, including common myeloid progenitors and megakaryocytic precursors, are BPIFB4-positive. To further study BPIFB4 expression in megakaryocytes, we handled MEG-01 cells, a megakaryoblastic cell line which is a useful *in vitro* model for studying megakaryopoiesis and platelets’ formation. Indeed, MEG-01 cells have morphological and phenotypical features resembling those of megakaryocytes, spontaneously release platelet-like particles (PLPs), and can be differentiated by PMA and TPO into mature megakaryocytes. Flow cytometry analysis showed the presence of BPIFB4 in naive MEG-01, and a significative increase in the percentage of BPIFB4^+^ cells upon MEG-01 differentiation (Fig. 3B). To deepen the biological value of BPIFB4 expression in differentiated MEG-01, we evaluated the impact of the overexpression of human wild type (WT)- and LAV-BPIFB4 isoforms in MEG-01 cells. Interestingly, the lentivirus-mediated overexpression of human LAV-BPIFB4 isoform, but not WT-BPIFB4 isoform was able in leading differentiated megakaryocytes to release more platelet-like particles (PLPs) (Fig. 3C).

Finally, we showed the presence of BPIFB4 not only in MEG-01 cells but also in PLPs (Fig. 3D).

Together, these data suggest that BPIFB4 is expressed in megakaryoblasts, increased in megakaryocytes and it is transferred to platelets. Thus, BPIFB4 emerges in multiple steps of the megakaryopoiesis process, and the longevity-associated variant might be functional to platelet release.

### Platelets fuel the M2 macrophage polarization by rhLAV-BPIFB4

**Fig. 4:**
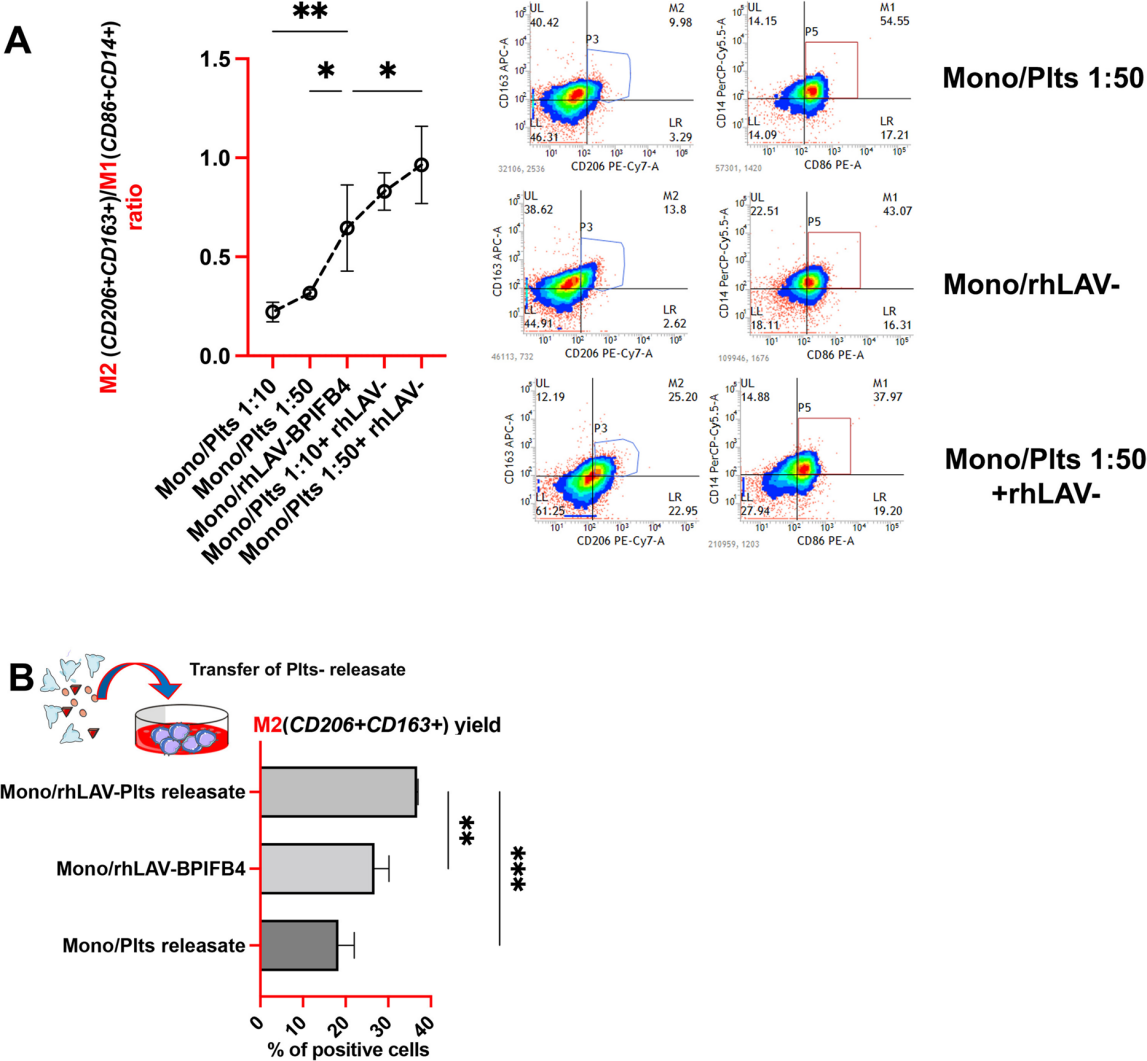
Platelets enhance the M2 macrophage skewing induced by rhLAV-BPIFB4. **A** Flow cytometric quantification of the M2(CD206 + CD163 +)/M1(CD86 + CD14 +) macrophage ratio (indicative of the macrophage polarization into an M2 or M1 macrophage-like phenotype). Donor’s monocytes were cultured for 7 days and led to differentiate into macrophages in the presence of platelets (at the ratio of monocyte to platelets of 1:50 and 1:10) and in the presence or absence of rhLAV-BPIFB4 (18 ng/mL). The result is representative of 3 independent experiments expressed as mean ± SD. Pairwise comparisons are reported (ordinary one-way ANOVA; *p < 0.05, **p < *0.01)*. The panel on the right side shows a representative, of three independent experiments, FACS dot plot analysis of CD86, CD14, CD206, and CD163 expression on macrophages’ surface following the differentiation process in the presence of the monocyte-platelets co-colture at the ratio of 1:50 and in presence or absence of rhLAV-BPIFB4 (18 ng/mL). **B** The bar graph shows the percentage of M2 macrophages (CD206 + CD163 + cells) harvested following 7 days in culture in the presence of rhLAV-BPIFB4 and/or the releasate from platelets stimulated with or without rhLAV-BPIFB4. The result is representative of 3 independent experiments expressed as mean ± SD. Pairwise comparisons are reported (ordinary one-way ANOVA; **p < 0.01, ***p < *0.001)*

At the translational level, not only the AAV-LAV-BPIFB4 constructs in vivo, but the use of the (rh)LAV-BPIFB4 in vitro has proven to be a useful therapeutic tool mainly by exerting immunomodulatory activity. Given the well-established M2-skewing activity of rhLAV-BPIFB4 [6, 9] and the functional relevance of the platelets in the mono-macrophage conditioning [16–18], then we evaluated whether platelets could modulate differentiation and polarization of monocyte-derived macrophages after 7 days co-colture in the presence or absence of rhLAV-BPIFB4 (18 ng/ml). As expected, stimulating monocytes with rhLAV-BPIFB4 induced differentiation to M2 macrophage by augmenting CD206 and CD163 expression levels with respect to both CD86 and CD14 levels (Fig. 4A). Remarkably, in the presence of platelets, rhLAV-BPIFB4-treated monocytes were more efficiently polarized toward the M2 phenotype, especially when monocyte-platelets co-colture was established at the ratio of 1:50 (Fig. 4A). When releasate from rhLAV-BPIFB4 stimulated platelets was exclusively added in colture, M2 macrophage polarization still occurred. This result highlights that the cell–cell contact is not essential for the achieved effects and that the early modulation of the platelets secretory profile mediated by rhLAV-BPIFB4 may be relevant to define the fate of mono-macrophage differentiation (Fig. 4B); which individual platelet factors, including systemic platelet factor 4 (PF4) remains to be determined.

## Discussion

According to the recent and unconventional role of platelets in mitigating the deleterious effects of aging [3], here we discovered that platelets may fuel most of the LAV-BPIFB4 protective effects. This is true for the vascular activity in vivo (Fig. 1D) and the favorable M2 macrophage polarization *in vitro* (Fig. 4). The platelet-dependence observed *in vivo* (Fig. 1) is consistent with the characterization of platelets as a new reservoir of BPIFB4 protein (Fig. 2). For the first time, herein we suggest that platelets could act as cellular messengers that modulate healthy aging achieved by LAV-BPIFB4. From a translational point of view, platelets derived from healthy LAV carriers (human) or LAV-treated (murine) ensure elevated plasma levels of circulating BPIFB4 (Figs. 2D and 1B). As BPIFB4 levels are considered a prognostic biomarker in prolonging health span, in determining COVID-19 and coronary artery disease (CAD) [12] severity, and the degree of carotid stenosis and intima media thickness in human patient cohorts [6], platelets could likely be the means of LAV-BPIFB4 therapeutic action. This is expected as platelets are key cellular players in those conditions, e.g., atherosclerosis, cardiomyopathy, and diabetic pro-thrombotic phenotype [19] in which the treatment with LAV-BPIFB4 resulted most effective to ameliorate both endothelial and cardiac function, mainly blunting inflammatory background [11, 12]. In a very recent study from Chen Li et *al.* [20], the authors highlighted the association between low platelet counts and immune dysfunction. Thus, future efforts to underpin the role of LAV-BPIFB4 in megakaryopoiesis and in the immune regulatory action of the platelets may be useful for human applications to prevent or treat the thrombocytopenia that would otherwise occur in several disease conditions, including sepsis, acute coronary syndrome, and in immunothrombosis.

Of note, in mice stressed with anti-platelet antibody, the pre-exposure of the whole animal to LAV-BPIFB4 gene therapy enhanced the survival of MK *in vitro* and/or increased the percentage of CD41 + cells among the primary BM cells (Fig. s1). This evidence could suggest compensatory megakaryopoiesis with a therapeutic value that deserves close attention. However, as putative caveat, we did not check the BPIFB4 transcript in platelets. Despite this, the presence of the protein in the highly specialized Megakaryocytes (MKs) suggests that platelet BPIFB4 can derive from those cytoplasmic proteins and granules that precursor cells accumulate for platelet function (Fig. 3A). Accordingly, western blot analysis for total BPIFB4 on whole cell extracts from murine platelet glycoprotein IIIa (CD61 +) positive cells, isolated through an immunomagnetic procedure on single-cell suspensions, highlighted a selective enrichment of BPIFB4 in large CD61 + megakaryocytes, compared to CD61- counterpart in bone marrow niche of AAV-LAV but not in AAV-GFP-injected control mice (Fig. s2). Consistently, we also found BPIFB4 to be highly expressed in megakaryoblastic leukemia MEG-01 cells (Fig. 3B). Here, surface FACS analysis of terminally differentiated MEG-01 showed a significant upregulation of BPIFB4 signal on the membrane from the breakdown of which platelets are usually generated. Accordingly, we reported a significant increase in PLP formation in LAV-BPIFB4 MEG-01 cells compared to WT-BPIFB4 or EMPTY MEG-01 counterpart (Fig. 3C). How the effects of genotype can influence the degree of platelets release both *in vitro* (Fig. 3C) and *in vivo* (Fig. 2D) remains to be examined, too. A possible explanation is the increased bioenergetic activity induced by LAV-BPIFB4 and that is required to make platelets. This is suggested by our recent paper demonstrating a potentiation of the mitochondrial metabolic pathways and the nucleolin-mediated ribosome biogenesis [12] in cardiomyocytes by LAV-BPIFB4 treatment. As a consequence, the LAV haplotype may maintain megakaryocyte viability, and thereby increase platelet shedding *in vitro* (Fig. 3C). Based on this hypothesis, one would expect that the overexpression of LAV-BPIFB4 would increase platelets count *in vivo*. Preliminary evidence show that a high platelets count is associated with LAV-BPIFB4 genotype with respect to WT and hetero-BPIFB4 patients of a small cohort of COVID-19 individuals in which BPIFB4 has been previously described to have a favorable prognostic relevance [21] (Fig. s3). This demonstrates, once again, the protective role of LAV-BPIFB4, when considering that thrombocytopenia has been reported in patients hospitalized with COVID‐19 and associated with worse clinical outcomes [22].

Finally, although our study describes the BPIFB4 release from platelets as beneficial, it is will also be important in the future to fully characterize the platelet activation response, including their aggregability in response to LAV-BPIFB4.

This will constitute adjunct value to an intriguing field of study based on novel LAV-BPIFB4 therapeutics.

## Materials and methods

### Human sample description

Human PRP samples were collected from 26 healthy donors. Seventeen PRP were secondly divided based on the different genotypes in hetero, WT, and LAV (etero: *n* = 6; WT: *n* = 8; LAV: *n* = 3) according to the variant of the protein BPIFB4 owned. All participants signed an informed consent for the management of personal anamnestic data and blood samples.

The collection of human BM biopsy was approved by the internal Ethics Committee of IRCSS MultiMedica: [n°PROT20/2010-Em3, “Rimodellamento e Disfunzione del Midollo Osseo nel Diabete Mellito”].

### Animal models

The overall objective of our in vivo study was to investigate the role of platelets in the AVV-LAV-BPIFB4 effects. To investigate this, we used C57BL/6 mice from Jackson Laboratories. Mice were randomly assigned to receive injections of either a control IgG or anti-CD42b to deplete circulating platelets after receiving AAV-LAV-BPIFB4 or EMPTY vector, as control. After 72 h of platelet depletion, mice were harvested, and the blood, lymphoid organs (bone marrow and spleen), and mesenteric aortas were collected. The Institutional Animal Care Use Committee of Neuromed Medical Center approved all animal experiments [n° 327/2023-PR]. Mouse colonies were maintained in the animal facility at IRCCS Neuromed, Pozzilli (IS), Italy. At week 10, mice were randomized into two groups and received either control antibody injections (polyclonal non-immune rat immunoglobulins) or a GPIb- alpha antibody (Emfret) injections to deplete circulating platelets (3 µg/g) through the caudal vein. At the end of treatment, mice were anesthetized with ketamine/xylazine, and euthanized by beheading to collect blood and tissue samples.

### *In vivo* gene therapy

The experimental procedure of both cloning and vector production and purification of BPIFB4 constructs have been detailed elsewhere [6].

Ten-week-old male C57BL/6 mice were anesthetized with 5% isoflurane in 100% O_2_ (delivery rate, 5L/min), and placed in a dorsal recumbent position on a homoeothermic blanket (N-HB101-S-402) to maintain body temperature at 37 °C. Anesthesia was maintained with 1% isoflurane in 100% O_2_ at 1.5 L/min, administered by means of a facemask connected to a coaxial circuit (Fluovac anesthetic mask). Vascular surgery was performed with the aid of a microscope at 2–10 × magnification. As previously described [6], femoral arteries were exposed and isolated circumferentially from the inguinal ligament to the knee. To avoid collateral damage to adjacent structures, the artery was carefully separated from associated nerves and veins with fine-tip forceps. After isolation of the arterial segment, a temporary clamp was closed around the proximal end of the artery to temporarily stop the blood flow. Subsequently, the distal end of the femoral artery was permanently occluded to perform a small incision on the superficial wall of the artery, using a syringe connected, through a PE10 – polyethylene tube, with a glass needle tip of 80 microns in diameter, to inject either 50 µl of saline containing AAV-GFP or AAV-LAV-BPIFB4 into the femoral artery. Viral titre was 1 × 10^13^ GC/kg for each experimental condition. After an incubation of 5 min, before carefully removing the syringe, we performed a permanent ligation just after the incision, and the clamp on the proximal femoral artery was removed to restore femoral blood flow and obtain systemic delivery. As support to the procedure’s success, evaluation of GFP revealed the expression of AAV9 (TBG-promoter) into the liver, thus confirming its systemic delivery. Mice remained anesthetized for 1 h, after which all received 100% O_2_ until recovery of righting reflex. Animal groups subjected to either control antibody injections (polyclonal non-immune rat immunoglobulins) or a GPIb- alpha antibody (Emfret) injections (3 µg/g), received a blood injection through the caudal vein (tail) after 24 h of AAV- delivery. Mice were sacrificed 96 h after surgery and vascular gene therapy. Mesenteric and femoral arteries were harvested and placed respectively on pressure and wire system for vascular reactivity studies.

### Mice tissue sampling and processing

Plasma and Peripheral Blood Mononuclear Cells (PBMCs) were extracted from whole blood of mice by Ficoll density gradient (Histopaque®-1077, Sigma-Aldrich). Plasma was employed to estimate the BPIFB4 levels dosage while PBMCs were stimulated *in vitro* with lipopolysaccharide (LPS) at a concentration of 1 µg**/**ml and then analyzed by flow cytometry.

CD61 + and CD61- cells were extracted from murine bone marrow through magnetically labeling and separation using CD61 MicroBeads (#130–109-678, MACS Miltenyi Biotec) following manufacturer’s procedures. CD61 positive and negative cells were further used for western blotting analysis.

### Cell lines and culture conditions

MEG-01 (ATCC® CRL-2021) were grown in humidified incubator at 37 °C and 5% CO_2_ in RPMI-1640 (Gibco®, Thermo Fisher Scientific) supplemented with 10% (v/v) fetal serum bovine (FBS, Gibco®, Thermo Fisher Scientific), 1% (v/v) penicillin–streptomycin (Aurogene), 1% (v/v) MEM non-essential amino acids (MEM NEAA, Gibco®, Thermo Fisher Scientific), and 1% (v/v) sodium pyruvate (Aurogene). Differentiated MEG01 were obtained upon 3 days pre-treatment with phorbol 12-myristate 13-acetate (PMA) 5nM and thrombopoietin (TPO) 100 ng/mL. Following the above-mentioned treatment, cells were collected for subsequent assays.

### Preparation of PRP from human and murine whole blood

Whole blood collected into vacutainer tubes containing 3.2% sodium citrate solution at the volume ratio 9:1 (BD Vacutainer System) was centrifuged at 2200 g for 30 s at room temperature (RT) to obtain platelet-rich plasma (PRP) by using aggregometer’s centrifuge system (Platelet Aggregation Profiler-PAP8, BIO/DATA CORPORATION, V 2.0 Optics). Platelets were counted using an automatic cell counter (LUNA Automated Cell counter, logos).

### Preparation of the monocyte-derived macrophages and platelets co-colture establishment

Peripheral blood mononuclear cells were extracted from the whole blood of a healthy donor by Ficoll density gradient (Cytiva Ficoll-Paque™ PLUS). After separation, PBMCs were washed and collected in MACS buffer (PBS 0.5% BSA, EDTA 2mM). CD14 + cells were harvested by immunomagnetic procedure following manufacturer’s protocol (human CD14 MicroBeads UltraPure, MiltenyiBiotec). Monocytes were seeded at 400.000 cells/mL in ImmunoCult-SF Macrophage Differentiation Medium (Stemcell Technologies) supplemented with 10% heterologous plasma. Monocytes were cultured for 7 days in the presence of autologous platelet-rich plasma at the ratio of monocyte to platelets of 1:10 or 1:50 with or without rhLAV-BPIFB4 (18 ng/mL). During the third day of culture, 50% of medium was replaced with fresh medium, and rhLAV-BPIFB4 (18 ng/mL) was added.

For macrophage conditioning with platelets’ releasate, PRP was diluted with Tyrode’s buffer (v/v 1:1) and centrifuged at 430 g for 15 min to obtain washed platelets. Platelets were resuspended in RPMI-free and stimulated with or without rhLAV-BPIFB4 (18 ng/mL) for 3 h at 37 °C. Platelets’ releasate was harvested after plate centrifugation at 1000 rpm. Monocytes were seeded and conditioned with platelets’ releasate considering monocyte to platelets ratio at 1:100 for 7 days in ImmunoCult-SF Macrophage Differentiation Medium (Stemcell Technologies) supplemented with 10% heterologous plasma.

### Production of lentiviral vectors and transfected EMPTY, WT, and LAV MEG-01 cell line

Lentiviral particles (Empty vector, WT- or LAV-BPIFB4) were generated as previously described [6]. Lentiviral particles were concentrated by ultracentrifugation (40,000 rpm for 2 h at 4 °C) and stored at − 80 °C until immediately prior to use. Lentivirus titration was performed by transducing HEK293T cells with concentrated particles in the presence of 4 µg/ml polybrene and measuring GFP expression after 3 days by flow cytometry. 500.000 MEG-01 cells were plated into a 12-well plate in RPMI medium and were infected with empty lentiviral vectors or particles encoding either WT- or LAV-BPIFB4 [at 3 multiplicities of infection (MOI)]. After 72 h, cells were selected with 1 µg/ml puromycin for 48 h.

### Immunohistochemistry on bone marrow and human PRP

For immunohistochemistry (IHC) staining, the following antibody was applied on human bone marrow tissue biopsies by using a routine immunoperoxidase technique: GeneTex C20orf186 antibody, rabbit polyclonal to KLH conjugated synthetic peptide derived from human C20orf186.

Briefly, decalcified human BM biopsy or femoral BM was embedded in paraffin, sectioned on a rotary microtome at 2 µm, and then dried, deparaffinized, and rehydrated. Enzymatic epitope retrieval was performed by microwave, boiled in sodium citrate buffer pH6. For Diaminobenzidine (DAB) reaction, endogenous peroxidase was blocked with H2O2 3%. Dako REAL EnVision/HRP, Rabbit/Mouse (ENV) was used for the detection of primary antibodies. The reactions were revealed by solution (1:50) of Dako REAL DAB + Chromogen and Dako REAL Substrate Buffer. Nuclei were stained with Mayer’s hematoxylin. All staining steps were performed at room temperature.

Morphometric analyses were performed on images captured with a digital camera at a final magnification of 40 × , using an image analysis software (Image proplus 4.0, Media Cybernetics, USA). Fresh human PRP was diluted in cytopath fixative (Diapath), to obtain a solution of 50 × 10^6^ platelets/ml. A monolayer of platelets was deposited on slides, in 20 mm diameter spots, using the semi-automatic instrument CytoPath Processor (Diapath). Samples were analyzed through immunohistochemical staining, using primary antibody anti-BPIFB4 (1:250), incubated at RT for 2 h, and the ultraView Universal DAB Detection Kit (Roche) for the detection. The kit consists of a cocktail of enzyme-labeled secondary antibodies. Negative control was prepared by incubating slides with only the secondary antibodies. Concurrently, slides were colored with GIEMSA staining, in order to underline the presence of platelets. Images were acquired with DMi1 inverted bright-field microscope (Leica), at 10 × magnification.

### Platelets count

Differentiated parental, EMPTY-, WT-, and LAV-MEG-01 cells were counted using an automatic cell counter (LUNA Automated Cell counter, logos) and two cell fractions were considered: platelet-like particles (PLPs, Ø 1–3 µm) and megakaryocytes (Ø 16–35 µm)**.** The ratio between MEG-01 cells and PLPs was carried out.

### Immunofluorescence on PRP

Tyrode’s buffer (Sigma-Aldrich) was added to the recovered PRP in a 1:200 ratio and centrifuged at 3000 g for 10 min to obtain the washed platelets. Afterwards, the platelets were resuspended in Tyrode’s buffer, loaded into a cytofunnel, and spotted onto the slide through the Cytospin (CENTURION-Scientific Limited) at 1200 rpm for 5 min. Platelets were fixed with 4% formaldehyde for 10 min at room temperature and washed three times with PBS (Gibco®, Thermo Fisher Scientific). Platelets were blocked and permeabilized using PBS with 5% Bovine Serum Albumin (PanReac AppliChem) and 0,005% saponin for 30 min at room temperature. Cells were incubated overnight at 4 °C with the following IgG primary antibodies’ mix: customized rabbit polyclonal anti-BPIFB4 (purchased from CliniSciences S.r.l.-Guidonia Montecelio – Italy; 1:100 in PBS 2.5% goat serum) and mouse polyclonal anti-P-selectin (Santa Cruz, 1:100 in PBS 2.5% horse serum). The next day, three washes of 10 min with PBS were carried out followed by a 30-min incubation at RT in the dark with the following secondary antibodies’ mix: Alexa Fluor 488-conjugate anti-mouse IgG (Vector Laboratories, 1:200 in PBS) and DyLight 549-conjugated anti-rabbit IgG (Vector Laboratories, 1:200 in PBS). Platelets were washed three times with PBS and nuclei were stained with DAPI (1:100 in PBS) for 20 min at room temperature in the dark. After five washes, slides were glycerol-mounted and images were acquired by using a confocal laser-scanning fluorescence microscope TCS SP5 (Leica Microsystems).

### Enzyme-linked immunosorbent assay (ELISA)

The BPIFB4 release was analyzed both on human PRP and plasma from mouse models. Human PRP in resting condition and upon stimulation with CaCl2 (22 mM for 40 min at 37 °C) or rhLAV-BPIFB4 (18 ng/mL for 40 min at 37 °C) were centrifuged at 13000 rpm for 5 min to collect the supernatants. BPIFB4 levels were determined using the Human Long palate, lung, and nasal epithelium carcinoma-associated protein 4 (C20orf186) ELISA kit (Cusabio CSBYP003694HU) following the manufacturer’s protocol. Briefly, supernatants were incubated for 2 h at 37 °C in the assay-coated microplate. After removing any unbound substances, a biotin-conjugated antibody specific for C20orf186 was added to the wells and incubated for 1 h at 37 °C. After washing, avidin-conjugated horseradish peroxidase (HRP) was added to the wells and incubated for 1 h at 37 °C. Following a wash, substrate solution was added and the consequent color development was stopped. Optical density was measured at 450 nm.

### Western blotting

CD61 + and CD61- cells from mouse bone marrow, platelets from human and murine PRP, and MEG-01 cells were washed with PBS (Gibco®, Thermo Fisher Scientific), harvested, and lysed in ice-cold RIPA lysis buffer (50 mM Tris–HCl, 150 mM NaCl, 0.5% Triton X-100, 0.5% deoxycholic acid, 10 mg/mL leupeptin, 2 mM phenylmethylsulfonyl fluoride, and 10 mg/mL aprotinin) as also detailed by Ciaglia et al. After centrifugation at 13,000 rpm for 20 min at 4 °C, in order to remove cell debris, proteins were quantified. About 30 µg of proteins were separated on 10% SDS-PAGE at 90 V for 1 h and at 120 V for another hour and then transferred to a nitrocellulose membrane. After blocking with 5% non fat dried milk powder (PanReacAppliChem) in Tris-buffered saline containing 0.1% Tween-20 (TBST) for 1 h at room temperature, the membranes were incubated overnight with the following primary antibodies: customized BPIFB4 (purchased from CliniSciences S.r.l.-Guidonia Montecelio – Italy) and β-actin (Abcam #4990 mouse mAb 1:50000). Immunodetection of specific proteins was carried out with horseradish peroxidase-conjugated donkey anti-rabbit IgG (Bio-Rad), using the enhanced chemiluminescence (ECL) system (Thermo Fisher Scientific) according to the manufacturer’s instructions and then exposed to X-ray films (Thermo Fisher Scientific). Western-blot data were analyzed using Photoshop software to determine the optical density (OD) of the bands. The OD readings of protein were expressed as a ratio relative to β-actin.

### Flow cytometry analysis

PBMCs of treated mice were stained with mAb against mouse CD86 (PO3.3; Miltenyi Biotec; 1:10), CD206 (C068C2; Biolegend; 1:50), and Ly6C (REA796; Miltenyi Biotec; 1:50). Human PBMCs were stained with mAb against human CD86 (REA968; Miltenyi Biotec; 1:50), CD206 (Miltenyi Biotec; 1:10), CD14 (HCD1-4; Biolegend; 1:50), CD163 (REA812; Miltenyi Biotec; 1:50). After 30 min incubation at 4 °C in the dark, cells were washed with staining buffer (PBS 2% fetal serum bovine, 0.01% sodium azide), centrifuged at 1800 rpm for 5 min, and resuspended in staining buffer for the FACS analysis. Instead, MEG-01 cells were incubated for 30 min at 4 °C in the dark with a primary customized anti-BPIFB4 (purchased from CliniSciences S.r.l.-Guidonia Montecelio – Italy, 1:100); then cells were washed with staining buffer and incubated with secondary PE donkey anti-rabbit IgG (Poly4064; Biolegend; 1:1000) for 30 min at + 4 °C in the dark. At the end of the incubation, the cells were washed as before and then resuspended in staining buffer for the FACS analysis. For each test, cells were analyzed using FACS Verse Flow Cytometer (BD Biosciences).

### Vascular reactivity study

Vascular reactivity studies were performed on second-order branches of the mesenteric artery, as previously described [6]. Quantification of vasomotor response was performed by a second individual who was blind to the genotype of the animal and/or the hypothesis that was being tested for each group. Vessels were isolated and dissected from fat and connective tissue in ice-cold Krebs solution and gassed with 95% O2 and 5% CO2. Subsequently, arteries were mounted on a pressure myograph in organ chambers with Krebs solution and treated with increasing concentrations of U46619 (10–9 to 10–6 M) to obtain a similar level of precontraction in each ring (80% of initial KCl-induced contraction). Caution was taken to avoid damage to the endothelium. Vascular r effect of LAV treatment was evaluated by assessment of the vasodilation response to acetylcholine (10–9 to 10–6 M). Vasorelaxation was expressed as a percentage reduction of U46619-induced contraction.

### Statistical analysis

In all experiments shown, statistical analysis was performed using the GraphPad Prism 6.0 software package for Windows (GraphPad software). For each type of assay or phenotypic analysis, the data obtained from multiple experiments were calculated as mean ± SD and analyzed for statistical significance using appropriate tests. In the analysis of variance (ANOVA) for multiple comparisons, *p*-values < 0.05 were considered significant; *p < 0.05, **p < 0.01, ***p < 0.001, and ****p < 0.0001.

## Supplementary Information

Below is the link to the electronic supplementary material.

Supplementary file1 (JPG 155 KB)

Supplementary file2 (JPG 1399 KB)

Supplementary file3 (JPG 133 KB)

## Data Availability

Data, materials, and protocols available on request by emails to the corresponding authors due to privacy/ethical restrictions.
