# Supplementary figures and images for: The longevity-associated BPIFB4 gene guarantees vascular homeostasis and immune protection through platelets
Source: GeroScience. 2024 Jun 17;46(6):6347–59. doi: 10.1007/s11357-024-01242-9 (PMC11493904; doi:10.1007/s11357-024-01242-9)

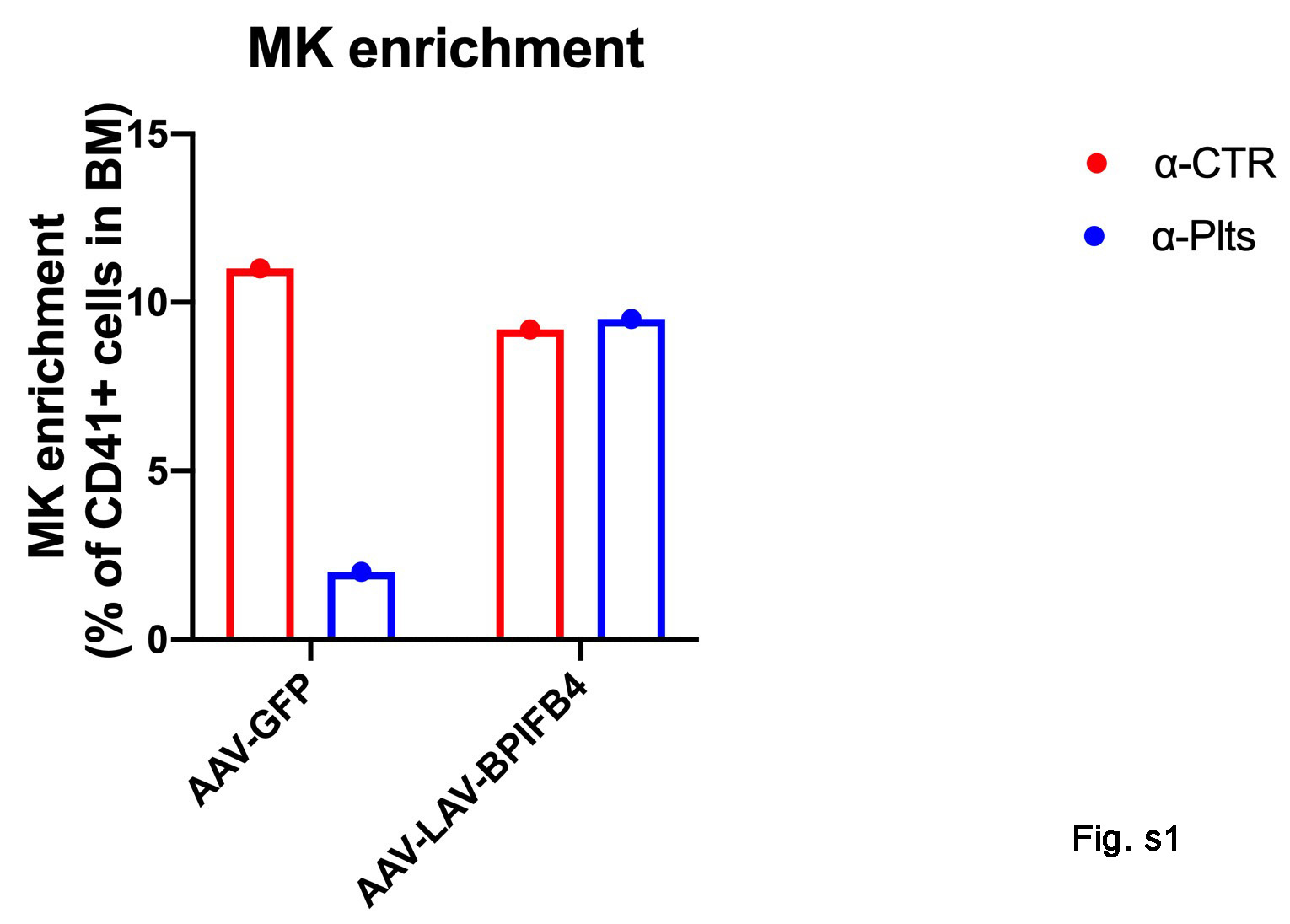

Supplement: Supplementary file 1 — Supplementary file1 (JPG 155 KB) [file 11357_2024_1242_MOESM1_ESM.jpg]

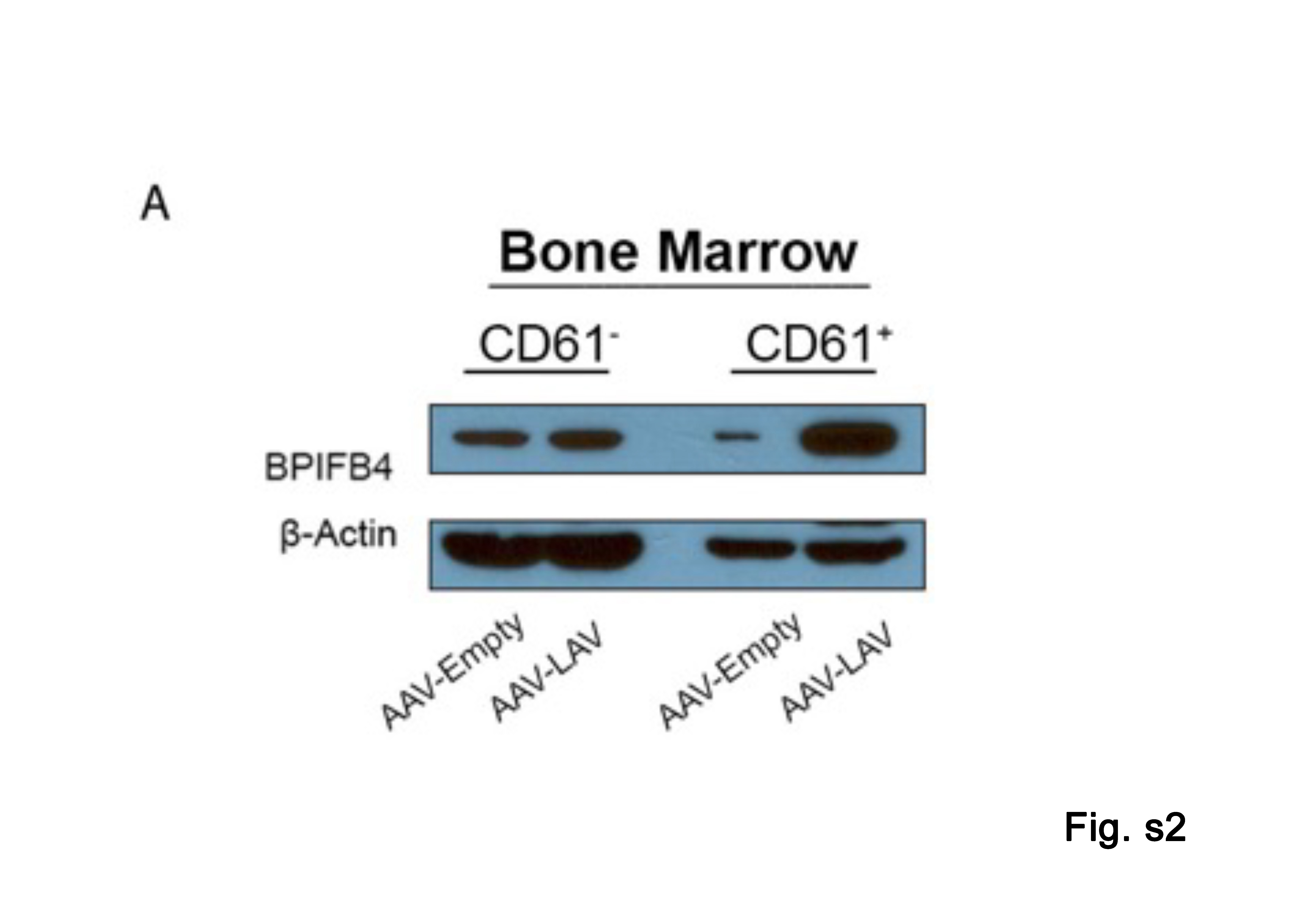

Supplement: Supplementary file 2 — Supplementary file2 (JPG 1399 KB) [file 11357_2024_1242_MOESM2_ESM.jpg]

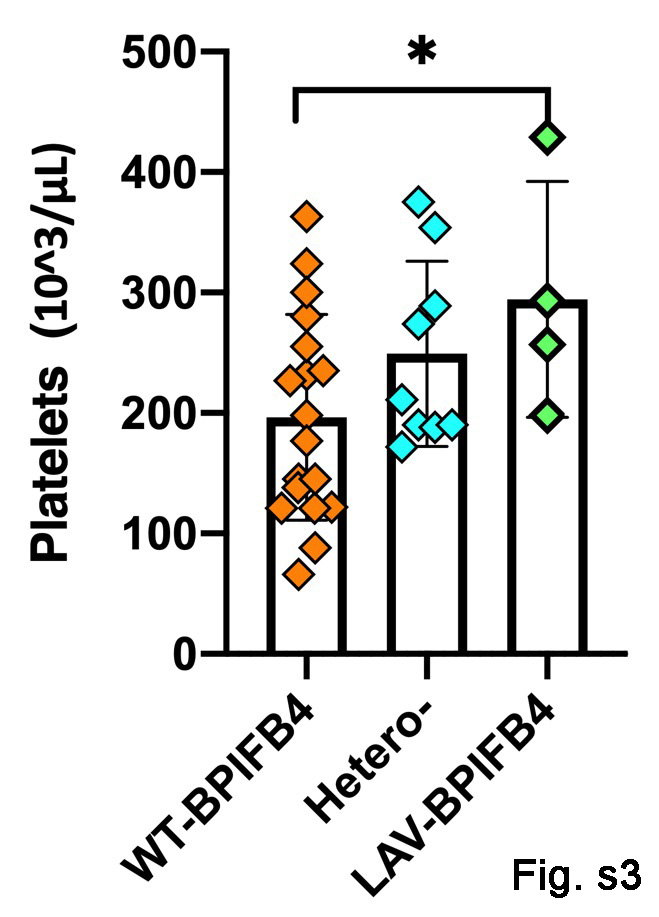

Supplement: Supplementary file 3 — Supplementary file3 (JPG 133 KB) [file 11357_2024_1242_MOESM3_ESM.jpg]
